# On Bloodletting in Pneumonia

**Published:** 1857-11

**Authors:** 


*On Bloodletting in Pneumonia. By Prof. Wunderlich.*

In the course of five years, there have been treated at the Leipsic Klinik 204 cases of pneumonia, of which number 36 (17.06 per cent) ended fatally; but if we abstract from these those cases which were brought to the hospital *in extremis*, and count only those which were actually treated there, there were then 190 cases with 11 deaths (11.57 per cent). Among the fatal cases, 3 were treated by bleeding, as were 44 of the cases that recovered, making the mortality of these so treated 6.38 per cent. These fatal cases were examples of pneumonia complicated with the disease of other organs.

In 114 of the patients, loss of blood occurred during the course of the pneumonia, whether from local or general bleeding, epistaxis or menstruations; and of this number 9 (including the 3 treated by bleeding) died, *i. e.* 7.89 per cent. In 76 cases, no loss of blood whatever occurred during the progress of the case, and of these 13, or 17.10 per cent died, not including persons brought in agony, and who had not in general been treated by bleeding. Thus it results that—1. In cases in which there was loss of blood in general the mortality was 7.89 per cent. 2. In those in which venesection had been employed 6.38 per cent. 3. In those in which a complete conservation of blood took place a mortality of 17.10 per cent.

The author enters into an elaborate comparative statement of the influence which the loss of blood exerts upon the time and mode of termination of the fever and of the commencement of the healing process. Pneumonia, he observes, possesses, in the vast majority of cases, the peculiarity of commencing with very determinate symptoms (severe chills, unequal distribution of the blood, and rapid increase of the objective temperature of the trunk), which are immediately followed by acute continued fever (increase of temperature, rapidity of pulse, etc.). In favorable cases, there is this further peculiarity, that at about the period of the completion of the exudative process (cessation of increased dulness on percussion, and of the bloody sputa) the febrile symptoms rapidly disappear, the delirium alone continuing awhile if it has been very violent. In this respect, pneumonia approaches the eruptive fevers, and forms a contrast to other inflammatory diseases, as abdominal typhus, pleurisy, peritonitis, meningitis, bronchitis, etc. Wishing to avoid the ambiguity which would ensue upon the adoption of the word crisis, the author designates this passage of the economy from a feverish to a feverless state, *defervescence.* It is no accidental occurrence, but a process which is sometimes rapid, sometimes slow, and may be complete or incomplete, protracted, uninterrupted, or remittent. A rapid defervescence is decisive for the quick convalescence of the patient; but, while cases in which it is remittent are rare, yet, when it is protracted, uninterrupted, it is of bad augury for the patient, even when the disease is slight.

As a standard for judging the effects of therapeutical agents upon the period of defervescence, the Professor first selects 32 cases treated by expectation, and in which the exact time of its commencement was noted. Taking 10 of severe and 10 of the medium cases, the defervescence commenced at the seventh or eighth day; but, taking the entire number, in adding 12 slight cases, it occurred at the sixth or seventh day. Judging from 9 cases which came under his notice (2 of menstruation and 7 of epistaxis), spontaneous bleeding proved rather favorable, as the improvement dated from the appearance of the bleeding.

Local without general bleeding was followed by recovery in 36 cases. In 26 it was employed either alone or in conjunction with medicines, such as digitalis or ipecacuanha, which exert no appreciable effect in expediting the period of defervescence, and in 10 it was combined with tartar emetic, which does exert an effect of this kind. Of the first series, rapid defervescence took place in 7 slight and medium cases in from the third to the sixth day, and in 19 bad cases it varied from the second to the ninth day. In the 10 cases of the second series, it took place from the third to the seventh day.

In 39 cases, in which the commencement of the disease could be accurately ascertained, venesection was employed. First, in 18 of these it was employed on the first or second day. In 10 of these there was immediate arrest of the process; in 2, immediate arrest, with a somewhat slower continuance of improvement; in 5, a considerable diminution of fever, with a later but less considerable return, the fever ceasing in 4 cases on the sixth, and in 1 on the seventh day. In 1, no effect was produced, improvement following only after local bleeding. Secondly, in 21 the venesection was performed from the third to the fifth day; but in none of these cases was bleeding the only means employed. The results obtained even here contrasted very favorably with those obtained by expectative treatment. It was found that the conjunction of tartar emetic hastened the period of defervescence somewhat; that of local bleeding was scarcely of any effect, while the addition of digitalis was of no effect whatever.—*Med. Times and Gaz.*, June 5, 1857, from *Virchow's Archiv.*, 1856.

